# A Viral Vectored Prime-Boost Immunization Regime Targeting the Malaria Pfs25 Antigen Induces Transmission-Blocking Activity

**DOI:** 10.1371/journal.pone.0029428

**Published:** 2011-12-28

**Authors:** Anna L. Goodman, Andrew M. Blagborough, Sumi Biswas, Yimin Wu, Adrian V. Hill, Robert E. Sinden, Simon J. Draper

**Affiliations:** 1 The Jenner Institute, University of Oxford, Oxford, United Kingdom; 2 Division of Cell and Molecular Biology, Imperial College London, London, United Kingdom; 3 Laboratory of Malaria Immunology and Vaccinology, National Institute of Allergy and Infectious Diseases, Rockville, Maryland, United States of America; Burnet Institute, Australia

## Abstract

The ookinete surface protein Pfs25 is a macrogamete-to-ookinete/ookinete stage antigen of *Plasmodium falciparum*, capable of exerting high-level anti-malarial transmission-blocking activity following immunization with recombinant protein-in-adjuvant formulations. Here, this antigen was expressed in recombinant chimpanzee adenovirus 63 (ChAd63), human adenovirus serotype 5 (AdHu5) and modified vaccinia virus Ankara (MVA) viral vectored vaccines. Two immunizations were administered to mice in a heterologous prime-boost regime. Immunization of mice with AdHu5 Pfs25 at week 0 and MVA Pfs25 at week 10 (Ad-MVA Pfs25) resulted in high anti-Pfs25 IgG titers, consisting of predominantly isotypes IgG1 and IgG2a. A single priming immunization with ChAd63 Pfs25 was as effective as AdHu5 Pfs25 with respect to ELISA titers at 8 weeks post-immunization. Sera from Ad-MVA Pfs25 immunized mice inhibited the transmission of *P. falciparum* to the mosquito both *ex vivo* and *in vivo*. In a standard membrane-feeding assay using NF54 strain *P. falciparum*, oocyst intensity in *Anopheles stephensi* mosquitoes was significantly reduced in an IgG concentration-dependent manner when compared to control feeds (96% reduction of intensity, 78% reduction in prevalence at a 1 in 5 dilution of sera). In addition, an *in vivo* transmission-blocking effect was also demonstrated by direct feeding of immunized mice infected with Pfs25DR3, a chimeric *P. berghei* line expressing Pfs25 in place of endogenous Pbs25. In this assay the density of Pfs25DR3 oocysts was significantly reduced when mosquitoes were fed on vaccinated as compared to control mice (67% reduction of intensity, 28% reduction in prevalence) and specific IgG titer correlated with efficacy. These data confirm the utility of the adenovirus-MVA vaccine platform for the induction of antibodies with transmission-blocking activity, and support the continued development of this alternative approach to transmission-blocking malaria subunit vaccines.

## Introduction

Despite considerable progress in efforts to control the transmission of malaria, the disease continues to cause approximately 225 million cases of clinical illness and nearly eight hundred thousand deaths each year [1]. The majority of severe disease in humans is caused by the *Plasmodium falciparum* parasite, transmitted exclusively by *Anopheles* mosquitoes. Insecticide treated nets (ITNs) can be used to interrupt the transmission cycle and have been shown in trials to reduce child mortality by 17% [2]. However ITNs do not appear to be as effective in protecting older children against febrile malaria [3]. Additional means of reducing malaria transmission thus remain urgently required to expand our armamentarium, including the development of an effective vaccine.

The most advanced malaria subunit vaccine, RTS,S/AS01, can induce 30–50% efficacy against clinical malaria, likely due to the generation of high titer antibodies against sporozoites [4]. This, along with the introduction of partially effective pre-erythrocytic control measures (e.g. ITNs, indoor residual spraying (IRS), artemisinin-based combination therapies (ACTs)), has led to a renewed interest in developing transmission-blocking vaccines (TBVs) – an approach that intercepts the *P. falciparum* life-cycle within the mosquito. This “community vaccination” approach would complement partially effective pre-erythrocytic control measures, and the development of an effective TBV is now widely viewed as essential for breaking the transmission cycle of malaria, especially following recent ambitious calls that the malaria community should aim for elimination or eradication [5].

A number of antigenic targets that induce transmission-blocking activity (TBA) in malaria have been investigated over the last 20 years [6], [7]. At present, the TBV immunogen which has been most widely studied and for which evidence is most compelling is the ookinete surface protein P25, although other parasitic (P48/45, P230, HAP2) and mosquito *Anopheles gambiae* aminopeptidase N (AgAPN1) antigens remain promising candidate targets [8], [9], [10], [11]. Antigen P25 in *P. falciparum* (Pfs25) is expressed in the macrogamete-to-ookinete stages of the parasite in the mosquito vector, and monoclonal antibodies against this antigen inhibit transmission [12]. This observation was subsequently translated into effective candidate subunit vaccines against malaria transmission in animal models [13], [14], [15]. Efficacy of transmission blockade was found to relate directly to ELISA titers, thus providing a relatively simple means of reliably estimating candidate vaccine efficacy [16]. Proof of principle of transmission blockade has also been established in humans using candidate *P. falciparum* and *P. vivax* P25 vaccines but unfortunately vaccines that have induced high titer antibodies were formulated in an adjuvant that appeared to be unsuitable for widespread human use, with an unacceptable incidence of side effects [17]. The development of a P25 protein vaccine has thus been hampered by the not uncommon issues surrounding the clinical suitability of various experimental adjuvants – including toxicity, low-level potency or lack of availability for commercial development, e.g. Freund's adjuvant, aluminium hydroxide, outer-membrane proteins and cholera toxin [18], [19], [20].

In recent years, the pre-clinical development of viral vectored blood-stage malaria vaccines has shown that moderately high level antibody responses can be induced by this alternative vaccine platform in mice [21], [22], [23], rabbits [24], [25] and rhesus macaques [26]. Antibody-mediated protection could be achieved against the *P. yoelii* mouse model of blood-stage malaria infection by using a priming immunization with an adenovirus vector followed by a booster immunization with the poxvirus vector MVA, and this approach targeting the blood-stage malaria antigens merozoite surface protein 1 (MSP1) and apical membrane antigen 1 (AMA1) has since entered Phase I/IIa clinical trials [27], showing this regime to be safe and similarly immunogenic in humans [28]. Viral vectored vaccines have a large insert capacity, allowing for entire proteins to be inserted under the control of well characterized promoters that drive high-level transgene expression, leading to potent vaccine immunogenicity. These viruses can be quickly expanded *in vitro* and purified for pre-clinical use, where they are administered intramuscularly in saline without the need for any chemical adjuvant [27], [29]. Immunization of mice with four doses of a recombinant human adenovirus 5 expressing the *P. vivax* homologue, Pvs25, led to antibody induction and TBA against *P. vivax* [30]. This success supports the use of a similar approach against *P. falciparum* using viral vectors expressing Pfs25.

The Pfs25 antigen is comprised of four EGF-like domains [31]. Allelic replacement studies suggest functional conservation between diverse *Plasmodium* species [32], [33]. Chimeric parasite lines of *P. berghei* expressing Pfs25 (TrPfs25Pb) and Pvs25 (Pvs25DR3) have been developed, and provide a suitable *in vivo* model for the assessment of vaccines targeting P25 [32], [33], [34], [35]. The approach of using transgenic rodent malarial parasites to assess the immune system's response to targets from a human malarial parasite has been additionally described in previous studies [36]. Such chimeric parasite lines provide a safe, cheap and more practical alternative to the use of non-human primate models for pre-clinical challenge studies of malaria vaccine efficacy. We have thus investigated the use of a similar transgenic model (Pfs25DR3) to test the efficacy of a heterologous prime-boost viral vectored *P. falciparum* Pfs25 vaccine regime *in vivo*. This approach to the assessment of anti-malarial transmission-blocking antibody activity is compared to the commonly used standard membrane-feeding assay (SMFA). In the SMFA, immune sera are mixed with *P. falciparum* gametocytes and the mixture is fed to mosquitoes via membrane feeders. Whilst this *ex vivo* assay provides useful information regarding the functional activity of antibodies, and is the “gold-standard” assay for assessing TBA, it is time-consuming, expensive and its use is limited to laboratories with access to *P. falciparum* culture facilities. As demonstrated by others [32], [33], [34], [36], [37] we have found chimeric parasites provide a simple *in vivo* challenge model, which can be used to investigate biological responses to parasites and to measure the efficacy of vaccines at the pre-clinical stage.

Here we evaluate, both *in vivo* and *ex vivo*, the ability of a heterologous prime-boost viral vectored Pfs25 vaccine regime to induce an anti-malarial transmission-blocking response. We show that immunization with AdHu5 Pfs25 elicits anti-Pfs25-specific IgG responses. Given the use of the AdHu5 vector may be limited in humans [38], a simian adenoviral vectored prime expressing Pfs25 was also developed. The use of a chimpanzee adenovirus 63 prime (ChAd63 Pfs25) was found to elicit comparable ELISA titers to AdHu5 Pfs25 at 8 weeks post-immunization. Immunization with AdHu5 Pfs25 prime, MVA Pfs25 boost (Ad-MVA Pfs25) also confers significant TBA – assessed by both the active immunization of mice challenged with transgenic *P. berghei* expressing Pfs25 (Pfs25DR3), and SMFA on cultured, sexually mature *P. falciparum* gametocytes. Our results demonstrate that a viral vectored, heterologous prime-boost regime against Pfs25 provides a promising adjuvant-free vaccine delivery platform, and as such could be considered as a potential tool for the development of future anti-malarial TBVs.

## Materials and Methods

### Ethics Statement

All procedures were performed in accordance with the terms of the UK Animals (Scientific Procedures) Act Project Licence and were approved by the University of Oxford Animal Care and Ethical Review Committee (PPL 30/2414) and the Imperial College Ethical Review Committee (PPL 70/6347). The Office of Laboratory Animal Welfare (OLAW) Assurance for Imperial College covers all Public Health Service (PHS) supported activities involving live vertebrates in the US (#A5634-01). Pooled human red blood cells and sera were purchased from the National Blood Service (UK). No ethics approval was therefore obtained for the use of human sera or red blood cells specifically but the Imperial College Ethical Review Committee reviewed all the procedures undertaken.

### Virus generation

The vaccine antigen used was encoded by *pfs25* from the NF54 isolate (GenBank X07802), commenced at amino acid 22 (αα) (alanine) and truncated prior to the transmembrane domain at αα 193 (threonine). To remove potential N-glycosylation sites, asparagines at αα sites 112, 165 and 187 were substituted with glutamine. The final antigen insert was codon optimized for mammalian expression and synthesized by GeneArt GmbH (Regensburg, Germany), and fused in frame to the human tissue plasminogen activator (tPA) leader sequence (GenBank Accession No. K03021) [21]. Insertion of the gene encoding Pfs25 into human adenovirus serotype 5 (AdHu5), chimpanzee adenovirus 63 (ChAd63) and modified vaccinia virus Ankara (MVA) was performed using methods previously described for other antigen inserts [21], [25]. Control AdHu5 and MVA vectors expressing GFP have been described previously [22].

### Pfs25 ELISA

ELISAs against Pfs25 were performed according to a standardized protocol [39] using a reference standard [40]. Protein antigen was provided by Dr Y. Wu (NIAID, NIH, USA). Test sera were diluted to 1∶100 or 1∶1000 in dilution buffer. Naïve sera were diluted to 1∶100 in dilution buffer and applied to 8 wells per plate. Positive control sera were provided by Dr Y. Wu (NIAID, NIH, USA) and were serially diluted on each plate to provide a standard curve. The ELISA units of this reference positive control sera was taken to be 14700. All sera (except naïve) were applied in duplicate and diluted 3-fold. Positive reference sera were applied to all plates. Plates were read after 20 minutes incubation in the dark at room temperature (RT). Reference sera diluted 1∶14700 gave an expected absorbance at 405 nm (OD405) of 1.0 at this time-point. A standard curve was prepared for each plate using the reference sera. The curve was plotted using a 4-parameter equation and plates were only accepted for further analysis if R^2^>0.998. The reference serum reading was taken to be at 14700 ELISA units. Using the OD405 values of test sera at the steep part of the curve (0.2–0.8), the ELISA units for each sample were calculated. The final result was the mean of the ELISA units calculated at a range of dilutions. Alternatively bound antibody isotypes were detected using biotin-conjugated rat anti-mouse IgG1, IgG2a, IgG2b or IgG3 follower by ExtrAvidin alkaline phosphatase conjugate using methods previously described [21]. The OD405 was read at a dilution of 1 in 500 and classed as no detectable antibody if the OD was then equal to or less than a non-immune naïve sample. A reference sample was also included on each plate as an internal positive control.

### Construction of Pfs25DR3

This transgenic line was constructed using previously published methods [32]. The targeting plasmid (kindly donated by Prof. N. Kumar, John Hopkins University, USA) was linearized by KpnI and NotI restriction digestion, and 10 µg was transfected into *P. berghei* ANKA 2.34 using the Amaxa human T cell nucleofactor kit as described previously [41]. Parasites were injected intraperitoneally (i.p.) into mice; 24 hours later mice were given drinking water containing 0.07 mg/ml pyrimethamine to select for drug resistant transfectants. Drug resistant parasites were taken through two successive rounds of selection, subjected to diagnostic PCR to confirm 5′/3′ integration and presence of Pfs25. Limiting dilution cloning was then carried out to create the transgenic parasite Pfs25DR3. Clones were again subjected to diagnostic PCR to confirm 5′/3′ integration, absence of Pbs25 and presence of Pfs25. The following primer pairs were used to detect 5′ integration of cassette, 3′ integration of cassette, presence of Pfs25 and absence of Pbs25 respectively.

(Primers: Int1 5′-CAT AGG GAT ATT TAC ATA CTC CTC-3′/Int2 5′-CAA ACA TTA TTT ACC ATA TCA TAT CC-3′/Int3 5′-CAA TGA TTC ATA AAT AGT TGG -3′/Int4 5′-GGT GGA AAA ACG CCC CCA AAT C-3′/Pfs25F 5′-AGT TAC CGT GGA TAC TGT ATG G-3′/Pfs25R 5′-CTG AAA AAG CAG TAC ATA TAG AGC-3′/Pbs25F 5′-GCG AGA TCT ATG AAT ACT TAT TAC AGT GTT TTT CTT-3′/Pbs25R 5′-GCG CCT AGA ATG ATA TTT GAA AAT ATT AGT AAA ATG AC-3′).

### Parasite maintenance

Routine parasite maintenance was carried out as previously described [42]. *P. berghei* ANKA 2.34 or *P. berghei* Pfs25DR3 parasites were maintained in 4–10 week old female Tuck Ordinary (TO) mice by serial blood passage (up to a maximum of 8 passages). Hyper-reticulocytosis was induced 2–3 days before infection by treating mice with 200 µl i.p. phenylhydrazinium chloride (PHz; 6 mg/ml in PBS; ProLabo, UK). Mice were infected by i.p. injection and infections were monitored on Giemsa-stained tail blood smears as described previously [42].

### Western blotting

*P. berghei* ookinetes from wild-type ANKA 2.34 and transgenic Pfs25DR3 were cultured and purified as described previously [33]. Samples were then analyzed by SDS-PAGE under reducing conditions, and western blot, using either pooled anti-AdHu5 Pfs25 (1∶500), or vector control pooled anti-AdHu5 GFP [22] serum (1∶500) followed by ECL™ sheep anti-mouse IgG (GE Healthcare) as a secondary antibody at a dilution of 1∶10,000. For loading controls, nitrocellulose membranes were probed using anti-TAT-1 (anti-α tubulin) antibody at 1∶10,000 (primary), and sheep anti-mouse IgG (GE Healthcare) at a dilution of 1∶10,000 as the secondary antibody.

### Immunofluorescence Assay

Expression and localisation of Pfs25 in the Pfs25DR3 transgenic parasite was confirmed via immunofluorescence assay on paraformaldehyde (PFA) fixed ookinetes. Briefly, Pfs25DR3 ookinete cultures were washed twice in ookinete media and then taken up in a 1∶1 mixture of ookinete medium and fetal bovine serum [8]. Smears were made and air-dried. Slides were fixed for 10 min in 4% PFA in PBS, and washed once with tris-buffered saline (TBS). Slides were blocked for 60 min in blocking buffer (10% v/v goat serum, 1% w/v BSA in PBS), following which, slides were washed once in TBS and incubated overnight at 4°C in a wet chamber with a reference mouse anti-Pfs25 antibody 4B7 (1 in 500) in 1% (w/v) BSA in TBS. The next day, slides were washed three times in TBS, incubated with Alexa Flour 488-labelled goat anti-mouse IgG (1∶1500 in 1% w/v BSA in TBS) for 45 min, washed three times in TBS, mounted with Vectashield/DAPI and analyzed by fluorescence microscopy.

### Active immunization

Groups of 15 mice were immunized for challenge experiments and divided randomly into groups of 5 mice for wild-type *P. berghei* challenge and groups of 10 mice for challenge with Pfs25DR3. For experiments to determine serology alone, groups of 10 mice were used. A single experiment to determine the immunogenicity of ChAd63 Pfs25 was performed using 25 mice in one group. Female BALB/c mice were 6–8 weeks of age at the start of the experiments (Harlan, UK). Mice were immunized intramuscularly (i.m.). Doses of vaccine used were 1×10^10^ viral particles (vp) of the AdHu5 or ChAd63 vaccines at week 0 and 1×10^7^ plaque forming units (pfu) of the MVA vaccines at week 10. Control mice were immunized with AdHu5 and MVA expressing GFP as an irrelevant antigen in place of Pfs25 (Ad-MVA GFP) [22]. Viral vectored vaccines were prepared in sterile, endotoxin-free PBS prior to immunization. Sera were collected at 2, 8, 10 and 12 weeks following the initial immunization. In a repeat experiment sera were collected at week 12 only. Following ChAd63 Pfs25 immunization sera were collected at week 8 only.

For challenge experiments, two groups of fifteen mice were immunized. One group was immunized intramuscularly (i.m.) with 1×10^10^ vp AdHu5 Pfs25 at week 0 and 1×10^7^ pfu MVA Pfs25 at week 10. The second (control) group was similarly immunized with Ad-MVA GFP control. At week 12 post-immunization, mice were PHz treated, and three days later infected i.p. with 10^6^ *P. berghei* ANKA 2.34 or *P. berghei* Pfs25DR3. In each group, 10 mice were challenged with Pfs25DR3, to assess anti-Pfs25 mediated transmission-blockade, and 5 mice were challenged with *P. berghei* ANKA 2.34, to assess potential non-specific transmission-blockade effects. Three days post-infection, >50 starved *A. stephensi* mosquitoes were allowed to feed on each infected mouse. 24 hours after feeding, mosquitoes were briefly anesthetized with CO_2_, and those that had not fed were removed. Mosquitoes were then maintained on fructose [8% (w/v) fructose, 0.05% (w/v) *p*-aminobenzoic acid] at 19–22°C and 50–80% relative humidity. On day 12 post-feeding, mosquito midguts were dissected, and oocyst prevalence and intensity recorded. For each mouse, oocyst numbers were counted and a calculation was made of mean oocyst intensity. For inhibition calculations this number was compared to mean oocyst intensity and infection prevalence in mice immunized with Ad-MVA GFP control. All care and handling of animals was in accordance with the Guidelines for Animal Care and Use prepared by Imperial College London.

### *P. falciparum* SMFA

For *P. falciparum* infections, erythrocytic stages of the NF54 isolate were cultured as described previously [43] followed by induction of gametocytogenesis [44]. Ten to 18 day old cultures demonstrating exflagellation of male gametocytes were then added to fresh human red blood cells (RBCs, group A, National Blood Service, UK) with heat-inactivated human AB serum (National Blood Service, UK) at a packed cell volume of ∼40%, and introduced into membrane feeders mixed with serum from mice harvested 12 weeks post-immunization at final dilutions of 1 in 5, 1 in 10 and 1 in 100. Sera from mice immunized with AdHu5 and MVA expressing GFP were used as a negative control. *Anopheles stephensi* mosquitoes of strain SDA500 were exposed to the membrane feeders for 25–30 min, and thereafter kept at 26°C and 80% relative humidity until dissection. Mosquito midguts were dissected 12 days post infection, stained with 0.5% mercurochrome and examined for oocysts using light microscopy. Transmission-blockade was assessed as reduction in mean oocyst intensity/prevalence in feeds supplemented with anti-Pfs25 sera, in comparison with negative control feeds at corresponding serum dilutions.

### Statistics

Statistical significance was analyzed using Prism (version 5). Antibody titers were log-transformed to enable parametric analysis. Where data were normally distributed parametric tests were used for data analysis. Where a mouse was tested over time, paired t-tests were used to compare ‘pre-boost’ (week 10) titers with ‘post-boost’ (time-points ≥12 week) titers. Parametric data were analyzed using a t-test or one-way ANOVA with Bonferroni's multiple comparison test. Correlations were tested using non-parametric Spearman rank correlations. Significance was taken as *P<0.05, **P<0.01 and ***P<0.001.

## Results

### Pfs25 specific total and isotype IgG responses following Ad-MVA Pfs25 immunization

**Figure 1:**
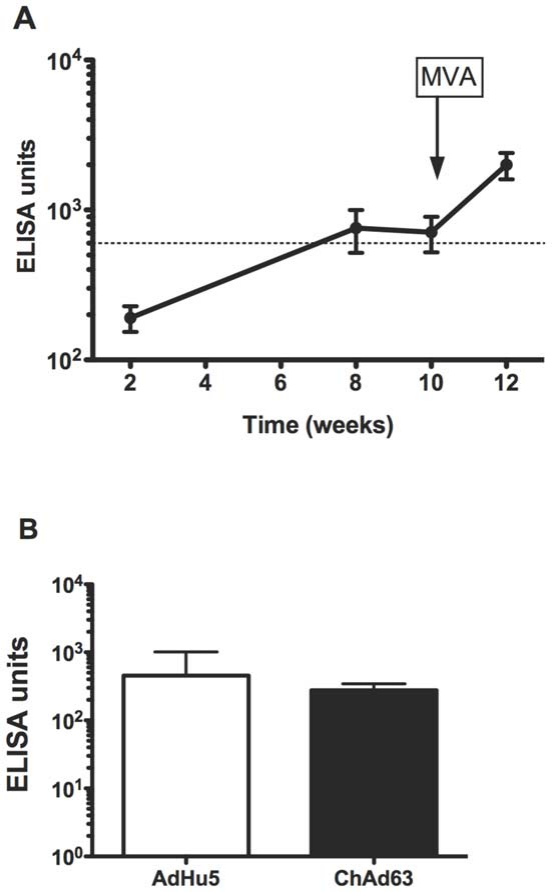
Pfs25 specific total IgG responses following Ad-MVA Pfs25 and ChAd63 Pfs25 immunization. *(A)* BALB/c mice were immunized with Ad-MVA Pfs25 (AdHu5 Pfs25 prime, MVA Pfs25 boost). Total IgG responses against recombinant Pfs25 protein were measured by ELISA in the serum of mice taken at the number of weeks following first immunization as shown. Mean ELISA titers ± sem (n = 10) are shown. Titers increased over time following the first immunization and were boosted following MVA Pfs25 administration at week 10 (arrow). The dotted line represents the titer required to provide 50% efficacy against *P. falciparum* in a SMFA using alternative vaccine platforms, according to previously published methods. *(B)* BALB/c mice were immunized with ChAd63 Pfs25 (n = 25) or AdHu5 Pfs25 (n = 10). Total IgG responses against recombinant Pfs25 protein were measured by ELISA in the serum of mice taken 8 weeks following a single immunization. Geomean ELISA titers ±95% CI are shown.

Ten BALB/c mice were immunized with 1×10^10^ vp AdHu5 Pfs25 at week 0 and 1×10^7^ pfu MVA Pfs25 at week 10 (Ad-MVA Pfs25). A second group of ten BALB/c mice were immunized with 1×10^10^ vp AdHu5 GFP at week 0 and 1×10^7^ pfu MVA GFP at week 10 (Ad-MVA GFP) as vector controls expressing an irrelevant antigen. Sera were taken at 2, 8 and 10 weeks following the first immunization and two weeks following the final immunization for measurement of total IgG responses against recombinant Pfs25 by ELISA. A further group of fifteen BALB/c mice were immunized in a repeat experiment in which sera were only taken at week 12. As found with previous mouse immunization studies for the blood-stage antigen MSP1 [21], [25], antibody titers increased over time following the recombinant AdHu5 immunization (figure 1a). There was a significant increase in titer from week 2 to week 10 (*P*<0.01) and a further significant increase in titers following the MVA boost (week 10 to week 12, *P*<0.05). The increase in titer from week 2 to week 12 was highly significant (*P*<0.001). The geometric mean titer at week 12 did not differ significantly between mice immunized in the replicated studies. Data from repeat studies combined, as statistical analysis permitted, to give a geometric mean titer at week 12 of 1664 (95% CI 1180–2345) ELISA units. The use of the standardized ELISA assay and a reference serum was performed to allow comparison of results obtained in this study with those analyzed using the same methodology [39]. At week 12, 96% of Ad-MVA Pfs25 immunized mice had antibody titers greater than 600 ELISA units, the level of polyclonal mouse serum expected to give 50% efficacy against *P. falciparum* in a SMFA [40]. No Pfs25 antibodies were detected in sera taken from GFP vector control immunized mice.

**Figure 2:**
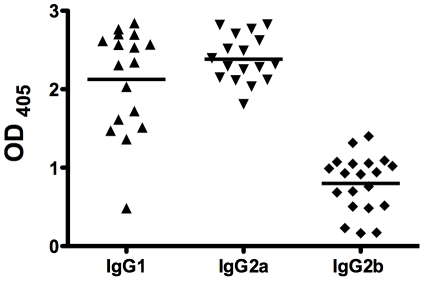
Pfs25 specific IgG isotype responses following Ad-MVA Pfs25 immunization. BALB/c mice were immunized with Ad-MVA Pfs25 as in figure 1a. Mice were immunized in two separate experiments and the data show pooled responses (n = 17–20). Individual isotype IgG responses against recombinant Pfs25 protein were measured by ELISA in the serum of mice taken at 2 weeks following final immunization. Individual and mean OD results are shown for samples measured at a dilution of 1 in 500. Positive (immune) and negative (non-immune) control sera were included in all assays. IgG3 was undetectable at this dilution.

To examine the utility of a simian adenoviral vector in a similar context, mice were immunized with ChAd63 Pfs25 and sera were taken at 8 weeks prior to MVA Pfs25 boosting. These samples were analyzed by ELISA and compared to the samples following AdHu5 Pfs25 immunization. There was no significant difference in titer from results at this time-point following AdHu5 Pfs25, by t-test of log-transformed data (*P* = 0.08, figure 1b). IgG isotype analysis following Ad-MVA Pfs25 immunization showed a mixed T helper type 1 (Th_1_)/Th_2_ IgG response with comparable levels of IgG2a and IgG1 (figure 2). Lower levels of IgG2b were also observed but IgG3 was not detectable. Similar IgG isotype profiles following Ad-MVA immunization have been observed in other murine studies with vectors encoding blood-stage malaria antigens[21], [24], [25].

### Generation and phenotypic analysis of Pfs25DR3

**Figure 3:**
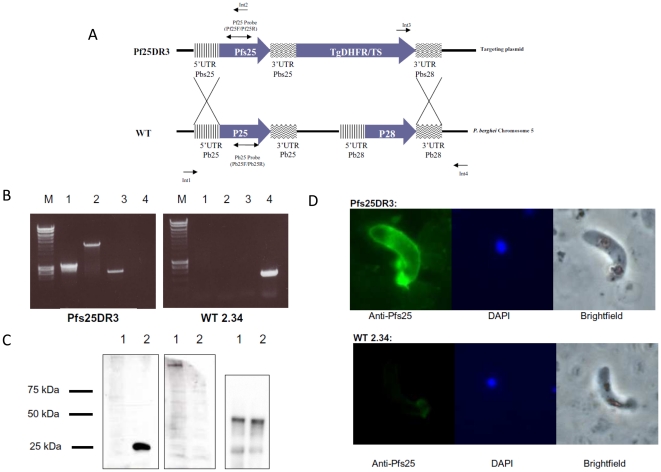
Generation and phenotypic analysis of Pfs25DR3. (A) Schematic representation of the construction of Pfs25DR3. The targeting plasmid was linearized by restriction digestion, and DNA was transfected into *P. berghei* ANKA 2.34. The *P. berghei* ORFs P25 and P28 were replaced with the *P. falciparum* gene sequence of Pfs25 and the selectable marker cassette (TgDHFR/TS). (*B*) Diagnostic PCR on cloned transgenic Pfs25DR3. (M: 500 bp marker; 1: 5′ integration, oligos Int1+Int2; 2: 3′ integration, oligos Int3+Int4; 3: Pf25 presence, oligos Pf25F+Pf25R; 4: Pb25 presence, oligos Pb25F+Pb25R. (C) Western blot analysis of Pfs25DR3 ookinetes. Purified ookinetes (5×10^7^ per lane) were hybridized with anti-Pfs25/GFP serum from Ad-MVA immunized mice. Loading control was carried out on stripped nitrocellulose membrane with anti-TAT-1 antibody at 1∶10,000 (primary), and anti-mouse IgG at 1∶10,000 (secondary). Lane 1: *P. berghei* WT 2.34 ookinetes; Lane 2: *P. berghei* Pfs25DR3 ookinetes. (D) Expression and localization of Pfs25 in the Pfs25DR3 transgenic parasite was confirmed via immunofluoresence assay on PFA fixed ookinetes.

To evaluate TBA *in vivo*, we created a transgenic *P. berghei* where the *P. berghei* open reading frames P25 and P28 were replaced with the *P. falciparum* gene sequence of Pfs25 and the selectable marker cassette (TgDHFR/TS). This was based on previously published chimeras [32], [33]. A schema of the process used is shown (figure 3a). Successful transfer of the selectable marker cassette was confirmed by PCR (figure 3b). An important caveat to the design and use of transgenic reporter parasite lines is that great care has to be taken to ensure that the anticipated antigenic target is the only potential target expressed by the reporter parasite, simple replacement of the homologous gene may not be adequate. In this case, the cross reactivity of antibodies to Pfs25 with Pbs28 was overcome by use of a construct that simultaneously removed both the homologous, and the paralogous, potentially ‘cross-reactive’ gene when the new target gene was inserted. This excluded the possibility of cross-reactive epitopes, conserved between Pfs25 and Pbs28 causing a non-vaccine specific transmission-blocking effect. The parasite is still fully viable throughout the entire lifecycle, as expected due to the (previously demonstrated) overlapping, partially redundant functions of P25 and P28 [45].

Serum harvested as described above was also assessed for its ability to recognize Pfs25 protein by Western blot (figure 3c) and immunofluorescence assays (figure 3d). Westerns on cultured and purified ookinetes from *P. berghei* Pfs25DR3, when probed with pooled anti-Pfs25 sera, produced a clear and distinct band at ∼25 kDa, indicating recognition of parasite expressed Pfs25. A corresponding band was not observed with purified *P. berghei* ANKA 2.34 wild-type (WT) ookinetes, indicating species-specific recognition of Pfs25 from the chimeric parasite. Blots on both WT 2.34 and Pfs25DR3 ookinetes using negative control serum from mice immunized with GFP vector control viruses were invariably negative. Immunofluorescence assay analysis using PFA fixed ookinetes confirmed the specific appropriate expression and localization of Pfs25 in the Pfs25DR3 transgenic parasite.

### Parasite infectivity following Ad-MVA Pfs25 immunization

**Figure 4:**
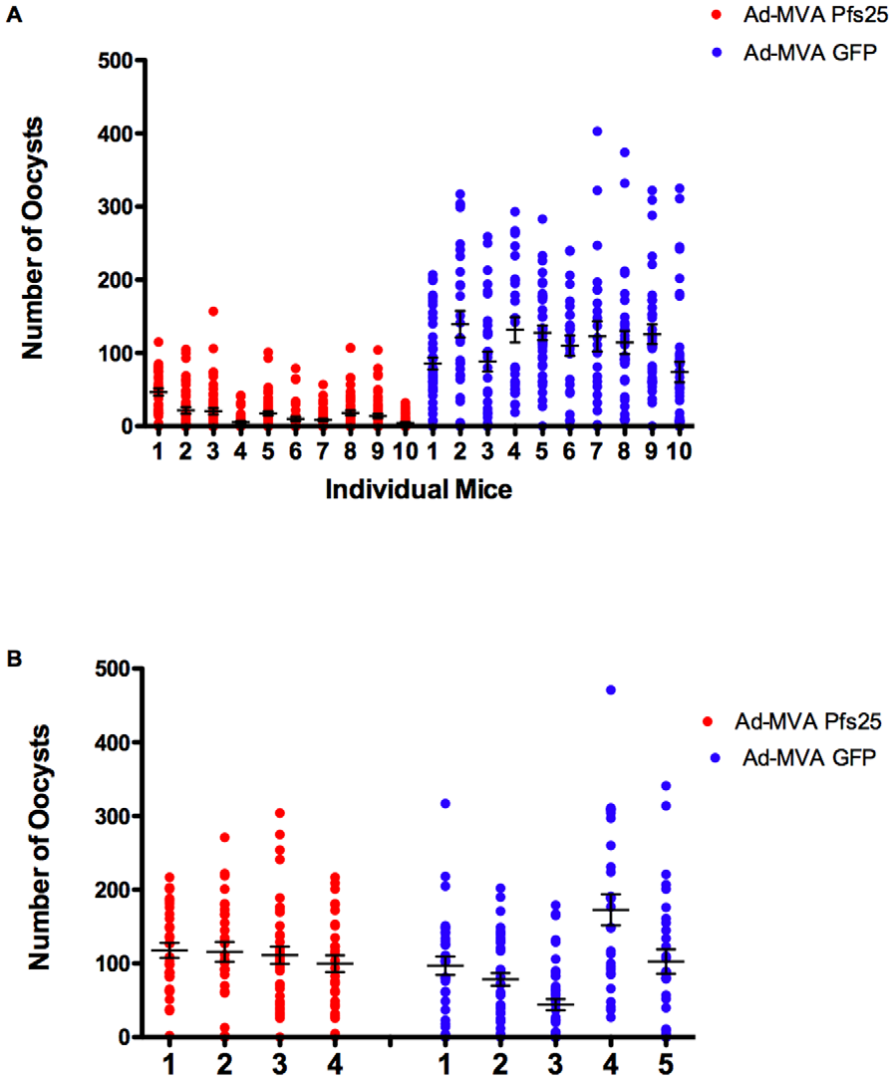
Parasite infectivity (per mosquito) following Ad-MVA Pfs25 immunization. BALB/c mice were immunized with Ad-MVA Pfs25 (red) as figure 1a or Ad-MVA GFP (blue) a GFP-encoding control. Mice were divided into two groups and infected with (*A*) Pfs25DR3 (10 per group) or (*B*) wild-type *P. berghei* (5 per group) and used to assess transmission to mosquitoes. Mosquito midguts were examined 12 days post-feeding. Data points represent mosquitoes that fed on individual mice. X-axis points represent individual mice. Horizontal lines indicate the mean number of oocysts for each mouse (+/− sem).

**Figure 5:**
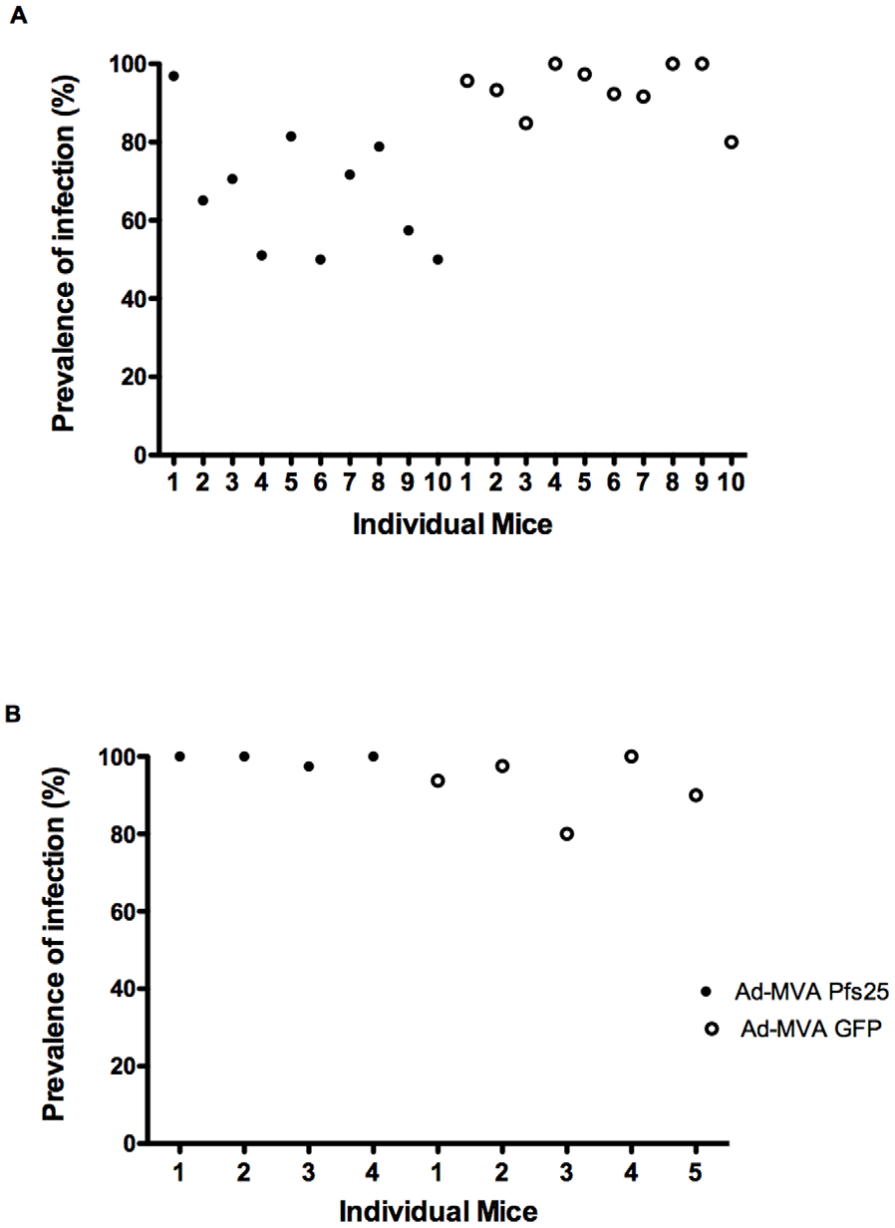
Parasite prevalence (per mouse) following Ad-MVA Pfs25 immunization. The same mosquitoes from figure 4 were analyzed for oocyst prevalence. Data points represent the prevalence of oocyst infection in mosquitoes that fed on individual mice, expressed as a percentage of total mosquitoes dissected.

**Figure 6:**
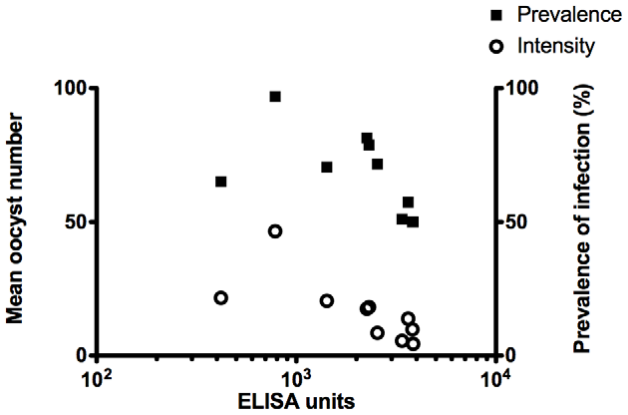
Total IgG antibody titer correlates with oocyst number. BALB/c mice (n = 10) were immunized with Ad-MVA Pfs25 and infected with Pfs25DR3 as previously (figures 4 & 5). Mean oocyst number (circles) and the prevalence of infection (squares) were calculated per mouse as shown in figures 4a and 5a. These data are shown plotted against specific anti-Pfs25 total IgG titer for each mouse.

Two groups of fifteen BALB/c mice were immunized as above with Ad-MVA Pfs25 or Ad-MVA GFP vector controls. Following the collection of sera from the Ad-MVA Pfs25 group for antibody ELISA, ten mice per group were subsequently infected with *P. berghei* Pfs25DR3, whilst five mice per group were infected with *P. berghei* wild-type (as controls to detect any potential non-Pfs25 dependent transmission-blockade). Direct feeds on immunized mice revealed significant inhibition of Pfs25DR3 oocyst intensity in mosquitoes fed on Ad-MVA Pfs25 immunized mice in comparison to controls. Mean oocyst number was reduced by 67% inhibition (*P*<0.001, figure 4a). Correspondingly, mean infection prevalence was reduced from 93% to 67%, a reduction in prevalence of 28% (*P* = 0.001, figure 5a). There was no significant reduction in wild-type *P. berghei* oocyst production when comparing mice immunized with Ad-MVA Pfs25 versus Ad-MVA GFP vector immunized controls (*P*>0.05) (figures 4b and 5b). A significant correlation was found between log-transformed total IgG and number of oocysts (Spearman rank correlation; *P* = 0.0008, r = −0.87, n = 10, figure 6, circles) and prevalence of infection (Spearman rank correlation; P = 0.01, r = 0.73, n = 10, figure 6, squares). No significant correlation was found between log-transformed IgG1, IgG2a or IgG2b, IgG1∶IgG2a ratio and number of oocysts or prevalence of infection.

**Figure 7:**
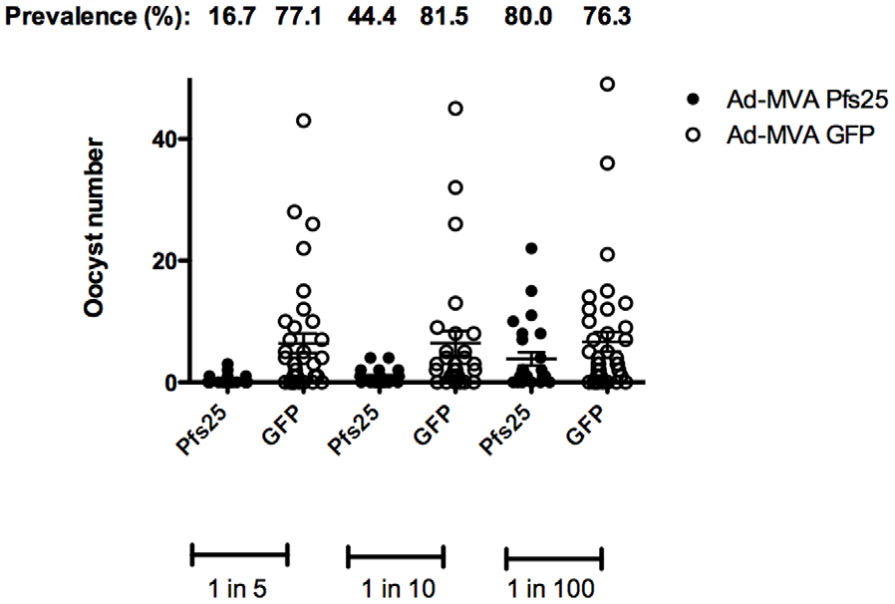
Parasite infectivity via membrane feeding asssay (per mosquito) following Ad-MVA Pfs25 immunization. Sexually mature *P. falciparum* NF54 cultures were mixed with anti-Pfs25 serum from a representative Ad-MVA immunized mouse (Pfs25) or negative control anti-GFP serum (GFP) at final dilutions of 1 in 5, 1 in 10 and 1 in 100. Data points represent the number of oocysts found in individual mosquitoes 12 days post feed. Horizontal lines indicate the mean number of oocysts (+/−sem).

To further determine the functional efficacy of antibodies induced by Ad-MVA Pfs25 immunization, a SMFA with *P. falciparum* (NF54 strain) was performed. Antisera from one representative mouse immunized with Ad-MVA Pfs25 and one Ad-MVA GFP control were tested for their ability to block oocyst development. Antiserum from the Ad-MVA Pfs25 immunized mouse showed significant transmission blocking activity, as measured by the fall in oocyst number in the mosquitoes which fed on antiserum from the Pfs25 immunized mouse compared to the control (figure 7). This mouse had a moderate ELISA total IgG titer of 1833 (geometric mean result for group 1664) so was representative of expected responses. Dose dependent inhibition was observed for both infection intensity (1 in 5: 96.0% inhibition; 1 in 10: 86.1%; 1 in 100: 42.3%), and infection prevalence (1 in 5: 78.3% inhibition; 1 in 10: 45.5%; 1 in 100: −4.8%). Statistically significant inhibition was demonstrated at serum dilutions of 1 in 5 (*P*<0.0005) and 1 in 10 (*P*<0.01) but not at a dilution of 1 in 100. Correlations with IgG were not assessed as serum from a single representative mouse was used in this assay.

## Discussion

Here we describe the successful induction of functional transmission-blocking antibody responses directed against Pfs25 following immunization with viral vectored vaccines recombinant for *P. falciparum* P25 (Pfs25). Immunization of mice with our previously reported AdHu5 prime – MVA boost regime [21], [23], [25] resulted in high, antigen-specific antibody titers that reacted with Pfs25 protein expressed by the Pfs25DR3 ookinete. ChAd63 Pfs25 prime appeared to be as effective as AdHu5 Pfs25 as assessed by ELISA. Previous studies using the same vaccine delivery platform have shown that IgG responses are maintained over time in mice, rabbits and humans [23], [25], [28], [46] – this is a particularly important consideration for TBV design, and it will be essential in future studies to assess maintenance of TBA over time as well as immediately following a booster immunization. Here we used a standardized ELISA in order that our results would be comparable with other studies [39] and were able to show that the levels of antibody titers achieved using this viral vectored regime at the peak of the response were similar to those recorded in studies of a Pfs25 protein-in-adjuvant vaccine following one or two immunizations in mice [19], [47]. Our vaccine regime thus enables the production of similar antibody levels without the need for potentially reactogenic adjuvants. Although higher titers, of approximately 5000–20000 ELISA units, have been reported elsewhere, the samples used in that study were specifically selected for their high titers [40]. Further comparison with other studies is limited by use of different ELISA methods [34], [48] or species [17].

Total IgG correlated inversely with oocyst intensity and infection prevalence, and the predominant IgG isotypes induced by Ad-MVA Pfs25 were IgG1 and IgG2a. Lower levels of IgG2b were also detected, but not IgG3. Murine studies of both DNA vaccines expressing Pfs25 and recombinant Pfs25 protein in Alhydrogel and CpG adjuvant have shown predominance of both IgG1 and IgG2a induction, although recombinant Pfs25 administered in Alhydrogel alone induced predominantly IgG1, with little IgG2a [47], [49], [50]. Recombinant Pfs25 administered in cholera toxin via the intranasal route led to production of comparable levels of IgG1, IgG2a and IgG2b but little IgG3 [48]. Antibody effector mechanisms targeting Pfs25 within the mosquito midgut could possibly involve ingested phagocytes and/or complement within the first five hours following blood meal uptake, alongside a more classical antibody neutralization mechanism [7], [51]. However it remains to be determined whether induction of specific IgG isotypes is important with regard to TBA mediated against the Pfs25 antigen.

Although strong antibody titers are of course desirable, the functional effect of antibodies on oocyst intensity/infection prevalence remains the “gold standard” readout to measure the effectiveness of a potential TBV. Here we have employed a proven transgenic parasite model [32] to compare the ability of sera from vaccinated mice to affect oocyst development in the SMFA as well as following live-feeding of mosquitoes on immunized and infected mice. In this study, mosquitoes fed on immunized mice showed a partial reduction in the level of a chimeric Pfs25DR3 infection, leading to a significant reduction in oocyst numbers in the fed mosquitoes of 67% and a reduction in infection prevalence of 28%. A direct correlation was observed between total IgG titer and oocyst numbers and prevalence of Pfs25DR3. Mlambo *et al.* have previously shown protection against a transgenic murine parasite expressing Pfs25 (TrPfs25Pb), by induction of Pfs25 antibodies following three DNA Pfs25 immunizations in mice, but were unable to explore the relationship between antibody titer and parasite infectivity given only a single high titer serum was used in the reported oocyst assays [32]. In a more recent study, they used the same parasite to assess the efficacy of a recombinant baculovirus expressing Pfs25 (AcNPV–Pfs25surf) and observed significant reduction in prevalence and oocyst numbers following three immunizations in mice [34]. The reduction in prevalence from approximately 80% to 54% (following intramuscular immunization of mice) or 65% (intranasal immunization) using AcNPV–Pfs25surf was comparable to that observed here.

Immunized mouse serum also showed significant transmission blocking activity (up to 96%) in a SMFA when fed to mosquitoes with *P. falciparum* and a substantial reduction in infection prevalence (78.3%). The use of a single mouse with a representative titer is consistent with other studies in which sera were pooled [30], [52]. Given serum from just one mouse was used for the SMFA it was not possible to correlate results with antibody titer, but the serum clearly showed more inhibitory effect when a higher concentration was used in the assay. Mlambo *et al.* observed a 50% reduction in *P. falciparum* oocyst number, as measured in a SMFA using mouse sera at an unspecified dilution [32]. The same group showed >90% inhibition using rabbit sera in a SMFA [34]. Arakawa *et al.* found a reduction in oocyst number to zero at dilutions of 1 in 2 to 1 in 32 using an inhaled recombinant Pfs25 protein vaccine, although the control oocyst count was relatively low [48].

Here we found a strong reduction in *P. falciparum* oocyst number at low serum dilutions of 1 in 5 to 1 in 10 (96% and 86.1% respectively), however the reduction in infection intensity and prevalence was less pronounced when using the chimeric *P. berghei* system but in agreement with this result, average oocyst intensities in the murine model were approximately ten-fold higher. Conversely, Mlambo *et al.* detected higher transmission block activity with chimeric challenge than with SMFA [32], as did Blagborough *et al.* [35]. It is not clear whether chimeric challenge or SMFA is more predictive of vaccine efficacy in humans, and vaccine efficacy measures in both systems will likely be affected by overall infection intensity. Interestingly, in one study it was found that the prevalence of infection when mosquitoes were fed via membrane feeders was approximately one third of that seen by direct feeding of mosquitoes on volunteers and no assessment was made of the impact of feeding method on oocyst number [53]. Further investigation is clearly required to directly compare live-biting and SMFA readouts in relation to vaccine efficacy in both the *P. berghei* and *P. falciparum* systems.

The clinical development of protein-in-adjuvant vaccines, including those targeting Pfs25 and Pvs25 [17], [54], has been limited by the side effects and uncertain safety profile associated with experimental adjuvants. On the basis of this and related studies [26], [30], we suggest viral vectored vaccines may therefore provide a promising alternative and clinically-relevant means of delivering comparable antibody immunogenicity for TBV candidate antigens. Indeed this vaccine platform has reliably induced antibody immunogenicity against the MSP1 and AMA1 blood-stage malaria antigens in mice, rabbits, rhesus macaques and humans [21], [22], [25], [26], [28], and further studies in mice and rhesus macaques have indicated that combining the protein and viral vectored subunit vaccine platforms may allow for the induction of even higher antibody titers [26], [46], [55]. The induction of extremely high titer IgG responses in humans by TBVs, that need to be maintained over the transmission season, may ultimately require the adoption of regimes that utilize combined vaccine delivery platforms. It is possible that a regime incorporating ChAd63-MVA Pfs25 and/or followed by Pfs25 protein would induce higher antibody titres and levels of protection *in vivo* than those observed here, and further work is ongoing in order to investigate such an approach.

Despite significant progress in recent years, there remains no licensed vaccine to protect against malaria infection or to help reduce transmission. Vaccines that act to prevent malaria transmission are likely to be of use in combination with existing malaria control measures (IRS, ITNs, ACTs) as well as other candidate vaccines that are currently in development against the liver- and blood-stages of infection [56], [57]. The data described here, generated using two different assay readouts, confirm the utility of the Pfs25 antigen to exert TBA and encourage the future clinical development of such viral vectored vaccine candidates that could be trialled alone or in combination with pre-existing protein vaccine candidates. Additionally, viral vectors encoding transmission blocking sexual- and mosquito-stage antigens could readily be combined, either in the same vector or as a mixture, with vectors encoding protective liver-stage and blood-stage antigens to provide a multi-stage vaccine. The ease of recombinant viral vector generation, especially for more difficult malaria antigens, also encourages the future screening of TBV candidate antigens utilizing this platform to generate antigen-specific IgG for testing in the SMFA. Such future studies could allow for the most efficacious TBV candidate antigen combination(s) to be taken forward for further clinical development.
